# Naringin ameliorates bone loss induced by sciatic neurectomy and increases Semaphorin 3A expression in denervated bone

**DOI:** 10.1038/srep24562

**Published:** 2016-04-25

**Authors:** Xinlong Ma, Jianwei Lv, Xiaolei Sun, Jianxiong Ma, Guosheng Xing, Ying Wang, Lei Sun, Jianbao Wang, Fengbo Li, Yanjun Li, Zhihu Zhao

**Affiliations:** 1 Institute of Orthopaedics, Tianjin Hospital, No. 122, Munan Road, Heping District, Tianjin TJ 300050, China; 2 Tianjin Institute of Orthopaedics in Traditional Chinese and Western Medicine, No. 122, Munan Road, Tianjin TJ 300050, China; 3 Graduate School of Tianjin Medical University, No. 22, Qixiangtai Street, Heping District, Tianjin TJ 300070, China

## Abstract

Naringin maintains bone mass in various osteoporosis models, while its effect on bone in disuse osteoporosis has not been reported. The present study explores whether naringin can prevent disuse osteoporosis induced by unilateral sciatic neurectomy (USN) and whether the Semaphorin 3A-induced Wnt/β-catenin signalling pathway is involved in the osteoprotection of naringin. Naringin dose-dependently prevented the deterioration of bone mineral density (BMD), trabecular structure and biomechanical strength in femur due to USN. Naringin increased bone formation but inhibited resorption, as indicated by bone-turnover markers in blood and urine and the histological staining of Osteocalcin (OCN) and tartrate-resistant acid phosphatase (TRAP) in femur. Semaphorin 3A (Sema3A) and active β-catenin protein decreased after USN and could be restored by naringin to the levels of the sham-operated rats. In addition, naringin *in vitro* promoted the differentiation of osteoblasts and inhibited osteoclastic differentiation. Our studies suggest that the down-regulation of Sema3A and the subsequent inactivation of Wnt/β-catenin signalling may be some of the mechanisms involved in USN-induced osteoporosis. Naringin could increase the expression of Sema3A and the activation of Wnt/β-catenin signalling to prevent disuse osteoporosis induced by denervation. Thus, naringin functions in bone maintenance and could be a promising therapeutic alternative in preventing disuse osteoporosis.

Semaphorin 3A (Sema3A), a secreted soluble protein, was originally identified as an axonal guidance chemorepellent with an essential role in nerve system (NS) development^1^ and regeneration following injury^2^; it belongs to the Semaphorin protein family^3^. Subsequently, various functions of Sema3A were reported outside of the NS, such as the regulation of immune systems, tumorigenesis and angiogenesis^4^,^5^,^6^. Recently, Sema3A was shown to play a pivotal role in skeleton development and in the maintenance of adult normal bone mass^7^,^8^,^9^. Hayashi *et al.* found that Sema3A, which is secreted by osteoblasts and osteocytes, could act as a local regulator of bone remodelling, promoting bone formation via inducing osteoblast differentiation mediated by the stimulation of the canonical Wnt/β-catenin signalling pathway and synchronously reducing bone resorption by inhibiting osteoclast differentiation^10^. The application of Sema3A *in vivo* could prevent bone loss induced by ovariectomy, accelerate the regeneration of bone defects and even increase the bone mass of normal adult mice^10^. Fukuda *et al.* later revealed that Sema3A, when expressed by sensory neurons innervating bone, exerted a more pronounced osteoprotective effect than did local Sema3A that was generated by bone cells. The knockdown of Sema3A specifically in neurons led to the deterioration of bone mass accompanied by a dramatic decrease in sensory nerves innervating bone^11^. Thereby, Sema3A could affect bone integrity directly by acting on bone cells and indirectly via the sensory nerve system.

Immobilization induced by the denervation of lower extremities has been widely used to induce experimental disuse osteoporosis in rodents^12^,^13^,^14^. Compared to the baseline value before sciatic lesion in a previous study, unilateral sciatic neurectomy (USN) led to a significant loss of bone mass in the cancellous bone of the ipsilateral femur and tibia, which rapidly progressed within four weeks after USN and then slowly progressed thereafter. Moreover, USN significantly decreased the expression of substance P, a neuropeptide that is released from sensory nerve terminals, in an ipsilateral denervated tibia four weeks after sciatic lesion, and subsequent treatment with substance p receptor antagonist further reduced the BMD of a denervated tibia that was already osteoporotic^15^. Because nerves innervating bone and neuropeptides within the bone microenvironment play an important role in the regulation of bone homeostasis^16^,^17^, it is hypothesized that, in addition to immobilization, the disturbance of peripheral neuron-derived signals also contributes to osteopenia induced by peripheral nerve neurectomy.

Naringin, a polymethoxylated flavonoid glycoside, is the main active constituent of citrus fruits, promoting the differentiation and proliferation of osteoblasts via the up-regulation of BMP-2 mediated by the activation of the PI3K-Akt signal pathway and by the enhancement of AP-1 binding to the promoter of BMP-2^18^, as well as enhancing the differentiation of bone marrow mesenchymal stem cells into osteoblasts^19^. In addition, naringin has an antagonistic effect on RANKL-induced osteoclastogenesis^20^ and induces osteoclast apoptosis via a mitochondria-dependent apoptotic pathway^21^. Moreover, naringin abrogates the bone loss of rodents induced by ovariectomy^22^, retinoic acid^23^ and glucocorticoid^24^. Naringin positively affects the bone qualities of senescent rats and orchidectomized rats^25^,^26^. Naringin can also activate the estrogen receptor^27^. However, the mechanism through which naringin plays an osteoprotective role is far from clear. In addition, it is unknown whether naringin has a positive effect on disuse osteoporosis and its corresponding mechanisms.

Because the sciatic nerve provides nerve fibres innervating the cancellous bone of the femur and tibia^15^ and because Sema3A derived from peripheral neurons and generated locally by osteoblasts and osteocytes plays a beneficial role in bone mass protection, we use here the transecting unilateral sciatic nerve model to investigate whether a change in the Sema3A signal is involved in bone loss induced by unilateral sciatic neurectomy (USN), which induces disused osteoporosis and is widely used in studies focusing on immobilization osteoporosis^12^,^28^, and whether naringin can prevent USN-induced osteoporosis and its corresponding mechanism.

## Materials and Methods

### Animals

All of the experimental procedures were approved by the Ethics Committee of Tianjin Hospital. The methods were carried out in accordance with the approved guidelines. Sixty healthy 6-month-old male Sprague–Dawley rats were obtained from the Experimental Animal Centre of Tianjin Hospital, the mean body weight of which was 260 ± 10 g. The rats were randomly assigned to five groups (n = 15 per group), four of which were treated with unilateral sciatic neurectomy (USN) and one with a sham operation (Sham group) as a control. The rats were maintained under a 12-h light–dark cycle and received a fixed amount of food and water ad libitum. For the USN model, the rats were firstly anesthetized with an intraperitoneal injection of ketamine hydrochloride. A dorsolateral incision was then made in the proximal thigh of the right hind limb to expose the sciatic nerve, 5 mm of which was excised. The proximal stump was deflected to prevent injured nerve regeneration. The rats in the Sham group received sciatic nerve exposure and mobilization without transection. The skin and subcutaneous tissues were then sutured layer by layer. The USN rats were divided into four groups: one with no further treatment (USN group) and three that were administered daily by gavage different doses of naringin (30, 100, or 300 mg/kg body weight, Sigma-Aldrich, St. Louis, MO, USA) dissolved in 0.9% saline solution. The USN group and the sham group were treated with an equivalent volume of 0.9% saline as a vehicle. The treatments were performed four weeks before and four weeks after USN, with the body weight recorded weekly.

To analyse the MAR using double-fluorescent-labelled bones, selective rats were injected intramuscularly with tetracycline (25 mg/kg) at 10 and 3 days before euthanasia. The bone mineral density (BMD) of the distal femurs ipsilateral to USN was measured the day before sacrifice. On the last day of administration, urine was collected by manual pressure-induced miction from overnight-fasted rats and stored at −80 °C. Upon necropsy, blood was collected from the heart by puncture exsanguination, and the serum was collected after centrifugation and preserved at −80 °C. The femurs and tibias in the denervated hind limbs were harvested and stored appropriately for further measurement.

### Bone mineral density (BMD) measurement

The BMD of distal femoral metaphysis *in vivo* was measured by dual-energy X-ray absorptiometry (DXA, Lunar-Prodigy; GE) as previously reported^15^. Briefly, the rats were placed in supine position after anesthetization with chloral hydrate (400 mg/kg, ip), with hind limb external rotation and hip, knee and ankle joints at 90° flexion.

### Trabecular microarchitecture analysed by Micro-CT

The trabecular microarchitectures of the distal femoral metaphysis were analysed using SIEMENS Inveon PET.SPECT.CT (SIEMENS, Berlin, Germany). The femurs were harvested and preserved in 70% ethanol. The region of interest (ROI) was defined as a region 25–125 slices from the distal femoral growth plate. The thickness of a slice was 21 μm, and the voxel resolution was 22 μm 3. Quantitative analyses of trabecular morphometric parameters, such as trabecula thickness (Tb.Th), trabecula number (Tb.N), trabecular separation (Tb.Sp), bone surface over bone volume (BS/BV) and bone volume to tissue volume (BV/TV), were conducted in VOI using the associated software package, with 3D images of the distal femurs reconstructed for visualization. All of the analyses were conducted by a blinded performer who did not know the treatments that the specimens received.

### Biomechanical property measurement

The harvested femurs were stored in 0.9% saline and used for mechanical testing as soon as possible. The mechanical properties of the femoral diaphysis and neck were performed as previously reported^29^ with the Electro-Force 3230 Bose System (BOSE, Minnetonka, MN, USA). After the soft tissues were removed, the shaft of the femur was fixed between two supporting points, with a distance of 20 mm. A load was vertically applied to the femoral midshaft, with a displacement speed of 0.01 mm/s at 100 Hz until the femoral shaft fractured. The load leading to midshaft fracture was recorded. The femurs that were tested for the mechanical strength of the femoral neck were cut at the midshaft level. The diaphysis that was left in the distal femur was fixed using methacrylate to lesser trochanter and maintained in a vertical position. Then, femoral head was loaded with a vertical compression, parallel to the long axis of the femur, with a preload of 1N and a speed of 0.01 mm/s, until the femoral neck fractured. The load-displacement curve of each bone was recorded, based on which the stiffness, maximal load and energy absorption of the femoral neck were analysed.

### Bone-turnover marker measurements

The frozen serum samples were thawed and immediately measured for the amino-terminal propeptide of type 1 procollagen (P1NP), an indicator of osteogenic activity, and cross-linked C-terminal telopeptides of collagen type 1 (CTX-1), an indicator of bone resorption activity, using corresponding ELISA Kits (BlueGene Biotech, Shanghai, China). In brief, 50 μl of standard or serum with sample diluent was added to a microtiter plate that had been pre-coated with the corresponding antibody and incubated at 37 °C for 30 min. After washing, 50 μl of enzyme conjugate was added to the plate, which was then incubated at 37 °C for 30 min. After washing, 50 μl of chromogenic reagent A and 50 μl of chromogenic reagent B were added to the microtiter plate. The plate was incubated at 37 °C for 10 min and protected from light. The reaction was terminated by adding 50 μl of stop solution. The absorbance was then measured at 450 nm. Frozen urine samples were prepared and used to measure urinary deoxypyridinoline (Dpd), a marker of bone resorption, similar to the protocol for serum using an ELISA Kit (BlueGene Biotech, Shanghai, China). The urinary creatinine (Cr) levels were measured to correct the Dpd levels.

### Histological and fluorescent analysis

After fixation in 4% neutral-buffered formaldehyde for 3 days and rinsing with tap water, the fluorescent-labelled distal femurs for histological analysis were dehydrated within successive ethanol. After clearing the used xylene, the specimens were embedded in polymethylmethacrylate (PMMA) without decalcification. The specimens were cut using a fine-diamond-coated saw (EXAKT 300 CP Band System, Norderstedt, Germany) along the sagittal plane of the distal femur, ground with a grinding machine (EXAKT 400 CP Micro Grinding System, Norderstedt, Germany) and then polished using number 4000 garnet paper to a thickness of 7 μm. The histological sections were observed using a fluorescence microscope (Nikon), and the mean tetracycline double-labelling interval was determined, which was then divided by the administration time interval to get MAR (μm/d).

### RNA isolation and quantitative real-time (qRT)-PCR analysis

The tibias of the denervated limbs were dissected and excised. After the soft tissues were removed, both sides of the tibia were cut out and the diaphysis was collected, which was then cut up and flushed with cold PBS to remove the bone marrow. The collected tibias were immediately stored in RNAstore reagent (ComWin Biotech, Beijing, China) and frozen in liquid nitrogen until mRNA measurement. The total RNA was isolated from the tibial diaphysis using Trizol reagent (Invitrogen, USA), reverse-transcribed to cDNA templates using the ReverTra Ace qPCR RT Kit (Toyobo Co. Ltd., Osaka, Japan), and amplified by quantitative the RT-PCR using SYBR^®^ Green Real-Time PCR Master Mix (Toyobo Co. Ltd., Osaka, Japan). The relative mRNA quantities of the transcripts were calculated according to formula 2^−ΔΔCt^ and normalized to that of the house-keeping gene β-actin. The mean relative transcript level was obtained in triplicate for each sample from five independent animals in each group. The primers were as follows: Sema3a F 5′ ACT GAC TCA CTG CTC CGA CT 3′ and R 5′ TAC CAA GGC TCT CTG TGA CT 3′; Nrp1 F 5′ TAC CCT CAT TCT TAC CAT CC 3′ and R 5′ CAC CTT CAT TCT CTC CAT CG 3′; β-catenin F 5′ AGA TCC TGA CCG AGC GTG GC 3′ and R 5′ CCA GGG AGG AAG AGG ATG CG 3; and β-actin F 5′ AGA TCC TGA CCG AGC GTG GC 3′ and R 5′ CCA GGG AGG AAG AGG ATG CG 3′.

### Western blot analysis

The tibias ipsilateral to USN were excised, and the soft tissues were removed. After both of the tibia extremities were cut off and the diaphysis was flushed with cold PBS to remove the bone marrow, the diaphysis was crushed in liquid nitrogen with a mortar and pestle and lysed in radioimmunoprecipitation assay (RIPA) buffer (Boster, Wuhan, China) with Phenylmethanesulfonyl fluoride (PMSF). The extracted protein samples were quantified using a BCA Quantitation Kit (Boster, Wuhan, China). Equivalent proteins (100 μg for Sema3A and its corresponding β-Actin; 60 μg for total β-catenin, active β-catenin and its corresponding β-Actin) isolated from the tibias of each group were separated on SDS-PAGE and transferred to a PVDF membrane (Boster, Wuhan, China). After blocking with 5% non-fat dry milk diluted in TBST at room temperature for 2 h, the membranes were incubated with goat polyclonal antibody against rat Sema3A (1:200 in 5% skim milk; Santa Cruz, CA, USA) or rabbit polyclonal antibody against rat β-catenin (1:200 in 5% skim milk; Boster, Wuhan, China) overnight. After being rinsed in TBST, the membranes were incubated with appropriate horseradish peroxidase-conjugated secondary antibodies (1:5000; Santa Cruz, CA, USA) at room temperature for 2 h and visualized with enhanced chemiluminescence (Santa Cruz, CA, USA). All the gels have been run under the same experimental conditions.

### Histochemical analysis

The femurs of the denervated limbs were fixed in 4% paraformaldehyde at 4 °C overnight and decalcified within 10% EDTA. After dehydration, the samples were embedded in paraffin and cut into slices with a thickness of 5 μm. The distal femurs were cut along the coronal plane and processed for the immunohistochemical staining of osteocalcin (OCN), haematoxylin-eosin (HE) staining, and tartrate-resistant acid phosphatase (TRAP) staining. In contrast, the femoral midshaft was cut along the transverse plane and processed for the immunohistochemical staining of Sema3A. TRAP staining was performed using a TRAP staining kit (Sigma, St. Louis, MO, USA) according to the manufacturer’s instructions, and counterstaining was performed with hematoxylin.

The protocol for immunohistochemical staining was as follows: after being incubated at 60 °C for 1 h, the sections were deparaffinized in xylene and rehydrated in ethanol. The activity of endogenous peroxidase was quenched by 3% H_2_O_2_ for 10 min. After the sections were rinsed, the antigen was retrieved by incubation within 10 mM boiling citric acid. The sections were blocked with 5% BSA at room temperature for 30 min and then incubated with primary antibodies against Sema3A (goat anti-rat, 1:100; Santa Cruz, CA, USA) or osteocalcin (mouse anti-rat, 10 μg/ml; Abcam, Cambridge, UK) at 4 °C overnight. After rinsing with PBS, the sections were incubated with biotin-coupled goat anti-rabbit secondary antibody, then with SABC (streptavidin–biotin complex) or biotin-coupled rabbit anti-mouse secondary antibody and then with SABC according to the manufacturer’s instructions (Boster, Wuhan, China). Signals were developed with 3,3-diaminobenzidine tetrahydrochloride kit (Boster, Wuhan, China). Nuclear counterstaining was performed with haematoxylin. Positive Sema3A or osteocalcin staining was observed using a Nikon NiE microscope (Nikon Optical, Tokyo, Japan).

### Differentiation of RAW 264.7 cells into osteoclasts

RAW 264.7 cells, a murine monocyte/macrophage cell line, were obtained from the Institute of Basic Medicine of Peking Union Medical College (Beijing, China) and grown in high-glucose DMEM supplemented with 10% foetal bovine serum and 1% penicillin–streptomycin within a humidified atmosphere and 5% CO2 at 37 °C. The differentiation of RAW 264.7 cells into osteoclasts (1 × 10^4^, in a 24-well plate) was induced by adding RANKL (100 ng/ml) to the medium for 5 days. Naringin was added to the differentiation medium for 7 days, with the ultimate concentration at 20 ng/ml. Osteoclasts were stained with TRAP using a TRAP staining kit (Sigma, St. Louis, MO, USA) in accordance with the manufacturer’s instructions.

### Cultures of MC3T3-E1 cells and ALP activity assay

MC3T3-E1 cells, a murine derived pre-osteoblastic cell line, were cultured in an alpha modification of Eagle’s minimum essential medium (α-MEM) supplemented with 10% FBS and 1% penicillin–streptomycin in a humidified atmosphere and 5% CO2 at 37 °C. The differentiation medium was prepared by adding 50 μg/ml ascorbic acid, 10 mM β-glycerophosphate and 1 × 10−8 M dexamethasone.

MC3T3-E1 cells that had been cultured in α-MEM with 10% FBS and 1% penicillin–streptomycin were seeded in a 24-well microplate (2 × 10^4^ cells/well). After 24 h, the medium was changed to the differentiation medium with or without naringin (0, 0.2, 2, 20 and 100 ng/ml) for another 7 days. After incubation for 7 days, the cells were harvested and washed with ice-cold PBS and then lysed by three cycles of freezing and thawing. Aliquots of supernatants from each group were used to quantitatively analyse the ALP activity using an ALP activity kit (Nanjing Jiancheng Biological Engineering Institute, China) according to the manufacturer’s instructions.

### Statistical analysis

The obtained data are expressed as the mean ± standard deviation (SD). Significant differences were analysed by GraphPad Prism V6.01 (San Diego, CA, USA). One-way ANOVA was used to determine the significant differences in the data among the five groups, followed by a Bonferroni multiple comparison test for pairwise comparison. Student’s t test was used for statistical analysis between the two groups. P < 0.05 was used for significance.

## Results

### ANOVA results

The ANOVA results indicated statistically significant differences among groups (USN group; 30 mg/kg, 100 mg/kg and 300 mg/kg naringin groups; and Sham group) for trabecular microarchitecture as analysed by Micro-CT (Tb.Th, *p* = 0.001; Tb.N, *p* = 0.000; BV/TV, *p* = 0.000; Tb.Sp, *p* = 0.000; BS/BV, *p* = 0.000), mechanical properties of the femoral neck (maximum fracture load, *p* = 0.001; energy absorption capacity, *p* = 0.002; stiffness *p* = 0.002), bone-turnover markers (P1NP, *p* = 0.000; CTX-1, *p* = 0.000; Dpd, *p* = 0.000), MAR (*p* = 0.000), ALP activity (*p* = 0.000), BMD (*p* = 0.000), results of qRT-PCR (Sema3a, *p* = 0.000; Nrp1, *p* = 0.000; β-catenin, *p* = 0.000), and western blot (Sema3A, *p* = 0.000; active β-catenin, *p* = 0.000), but not by the femoral midshaft maximum load (*p* = 0.3623). Detailed information of the multiple comparison results among all of the groups is shown below.

### Femoral trabecular micro-structure

**Figure 1:**
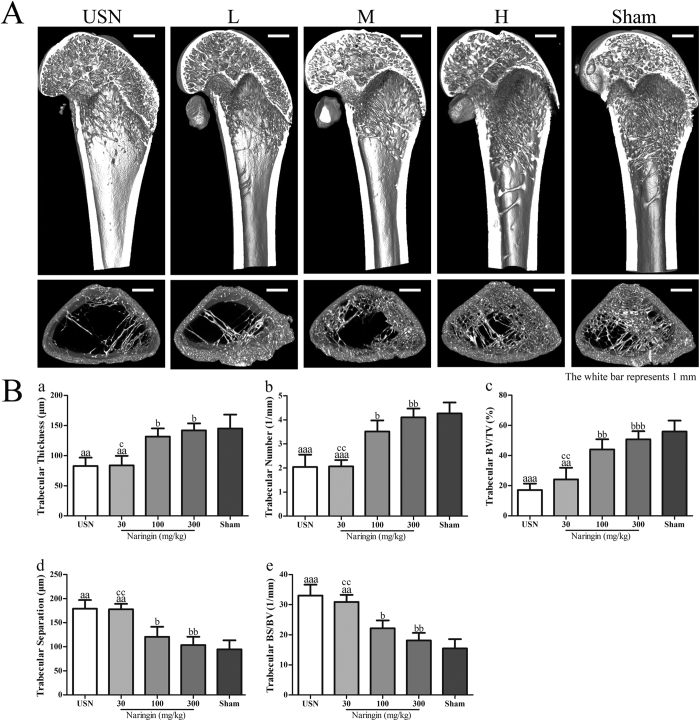
Effects of naringin on the protection of the trabecular microstructure as analysed by Micro-CT scanning. The region of interest (ROI) that was chosen for analysis was a region 25–125 slices away from the distal femoral growth plate at the distal femoral metaphysis. The thickness of a slice was 21 μm. (**A**) Representative 3D images showing the trabecular microarchitecture in the distal femoral metaphysis of each group. (**B**) Micro-structure parameters of the distal femoral trabecula as analysed from Micro-CT scanning data. Naringin at higher doses prevents the femoral trabecular micro-structure from deteriorating by immobilization at 4 weeks. L represents the 30 mg/kg Naringin group, M the 100 mg/kg Naringin group, and H the 300 mg/kg Naringin group. (a) represents the trabecular thickness (Tb.Th), (b) the trabecular number (Tb.N), (c) the trabecular bone volume fraction (BV/TV), (d) the trabecular separation (Tb.Sp) and (e) the surface/volume ratios (BS/BV). ^a^p < 0.05, ^aa^p < 0.01, ^aaa^p < 0.001 vs. Sham group. ^b^p < 0.05, ^bb^p < 0.01, ^bbb^p < 0.001 vs. USN group. ^c^p < 0.05, ^cc^p < 0.01, ^ccc^p < 0.001 vs. 300 mg/kg Naringin group. One-way ANOVA.

The Micro-CT scanning of the distal femur was analysed among all of the groups to evaluate whether naringin maintains trabecular micro-architecture deteriorated by immobilization. The representative three-dimensional reconstruction images of Micro-CT scanning from the distal femoral trabecular of the five groups with visible microstructural differences are shown in Fig. 1A. The data from the Micro-CT scanning demonstrated that Tb.Th, Tb.N and BV/TV in sciatic neurectomized rats dramatically decreased compared to those of the sham-operated rats (Fig. 1Ba–c; *p* < 0.01). In contrast, the values of Tb.Sp and BS/BV in sciatic neurectomized rats were much higher than those of the Sham group (Fig. 1Bd,e; *p* < 0.01). However, the preventive application of naringin at higher doses effectively attenuated the deterioration of all five parameters due to USN. Compared to those of the USN group, the trabecular Tb.Th, Tb.N and BV/TV increased significantly (Fig. 1Ba–c; *p* < 0.05 and *p* < 0.05, respectively), accompanied by decreased Tb.Sp and BS/BV (Fig. 1Bd,e; *p* < 0.05 and *p* < 0.01, respectively) in the 100 mg/kg and 300 mg/kg naringin groups. The five measurement indexes in the 300 mg/kg naringin group were restored to the levels of the Sham group (Fig. 1Ba–e; *p* > 0.05). Thus, naringin protects against USN-induced trabecular micro-structure failure.

**Figure 2:**
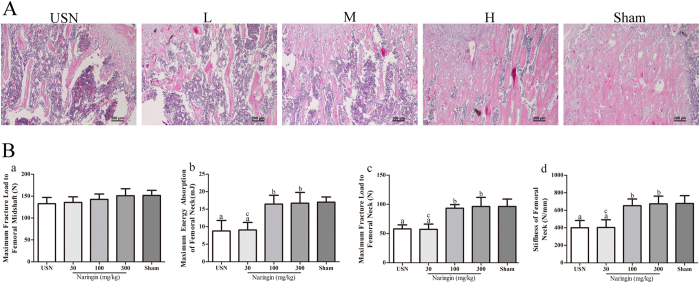
Pathological observation of the distal femoral metaphysis in each group as stained by HE (**A**). Effects of the prophylactic administration of naringin on the prevention of the deterioration of biomechanical properties of the ipsilateral femurs for 4 weeks after USN (**B**). The upper panel: A photograph of the longitudinal section at the distal femoral metaphysis shows that the preventative application of higher doses of naringin significantly alleviates the destruction of cancellous bone microarchitecture caused by USN (**A**). The lower panel: A three-point bending test was performed to assess the bone strength of the femoral shaft, while a compression test was conducted to determine that of the femoral neck. (a) The maximum load of femoral diaphysis, and (b–d) the energy absorption, maximum fracture load and stiffness of femoral neck (**B**). ^a^p < 0.05, ^aa^p < 0.01, ^aaa^p < 0.001 vs. Sham group. ^b^p < 0.05, ^bb^p < 0.01, ^bbb^p < 0.001 vs. USN group. ^c^p < 0.05, ^cc^p < 0.01, ^ccc^p < 0.001 vs. 300 mg/kg Naringin group. L represents the 30 mg/kg Naringin group, M the 100 mg/kg Naringin group, and H the 300 mg/kg Naringin group. Magnification ×100. One-way ANOVA.

To further confirm the effect of naringin on trabecular micro-structure, the bone histomorphometry in the distal femur ipsilateral to sciatic neurectomy was also examined by HE staining (Fig. 2A). The USN rats showed fewer and much thinner trabeculae than did the rats of the Sham group, together with a loss of reticulate structure and an increased number of free ends. However, following naringin administration at higher concentrations, the distal femoral trabeculae were greatly improved, with more connectivity, much wider trabeculae and less intertrabecular space compared to the USN group, especially in the 300 mg/kg naringin group.

### Mechanical properties of the femoral shaft and neck

Because the preservation of trabecular micro-structure contributes to bone strength dramatically, we next evaluated the biomechanical properties of the femur among all of the groups. To evaluate the mechanical strength of the femur, three-point bending loading was performed on the femoral midshaft to measure the maximum fracture loading, while a compression test was conducted to determine the maximum fracture loading, stiffness and energy absorption capacity of the femoral neck. After USN application for four weeks, no significant differences in the femoral midshaft maximum load were found between the USN group and the Sham group (Fig. 2Ba; *p* > 0.05). Treatment with naringin showed no further effect on the midshaft maximum load compared to that of the Sham group (Fig. 2Ba; *p* > 0.05). Nevertheless, the energy absorption capacity, maximum fracture load and stiffness of the femoral neck in the USN group significantly decreased compared to those of the sham-operated rats (Fig. 2Bb–d; all *p* < 0.05). The energy absorption, maximum fracture load and stiffness of the femoral neck in the naringin-treated groups at 100 mg/kg and 300 mg/kg were much higher than those in the USN group (all *p* < 0.05) but showed no significant differences with those of the Sham group (Fig. 2Bb–d; all *p* > 0.05). Thereby, naringin could improve and maintain the mechanical properties of the femoral neck under the condition of limb immobilization.

### Biochemical parameters and BMD of the distal femur

**Figure 3:**
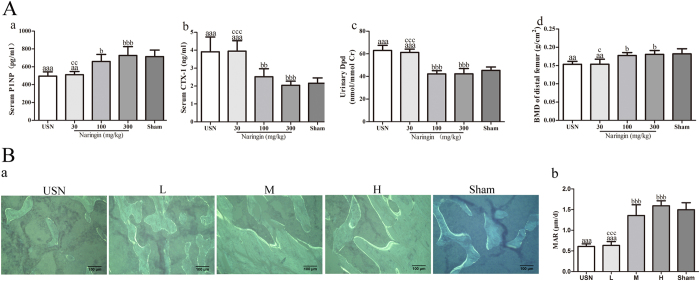
Effects of naringin on bone-turnover markers and BMD (**A**) and Effect of naringin on the bone formation in each group (**B**). The upper panel: Serum P1NP, an indicator of osteogenic activity, and serum CTX-1 and urinary Dpd, indicators of bone resorption, were measured in all of the groups using corresponding ELISA Kits. The BMD of the distal femoral metaphysis was analysed *in vivo* using DXA. Naringin significantly ameliorated immobilization-induced the decrease in serum P1NP (a) and increase in serum CTX-1 (b) and urinary Dpd (c). In addition, naringin maintained the BMD in the distal femur (d) (**A**). The lower panel: (a) Representative undecalcified sections at 4 weeks post-operation show the fluorescent-labelled bone sections through the injection of tetracycline; (b) Quantitative results of MAR reveal that naringin significantly attenuated the decreased MAR induced by neurectomy, n = 5 (**B**). ^a^p < 0.05, ^aa^p < 0.01, ^aaa^p < 0.001 vs. Sham group. ^b^p < 0.05, ^bb^p < 0.01, ^bbb^p < 0.001 vs. USN group. ^c^p < 0.05, ^cc^p < 0.01, ^ccc^p < 0.001 vs. 300 mg/kg Naringin group. L represents the 30 mg/kg Naringin group, M the 100 mg/kg Naringin group, and H the 300 mg/kg Naringin group. Magnification ×100. One-way ANOVA.

Because the appropriate bone remodelling activity contributes significantly to normal bone quality and integrity, we measured the bone-turnover markers P1NP and CTX-1 in serum and Dpd in urine. As illustrated in Fig. 3Aa, a significant decrease in the plasma P1NP was found in rats of the USN group, decreasing by 30.6% compared to the Sham group (*p* < 0.001). The intragastric administration of naringin at 100 mg/kg and 300 mg/kg dramatically increased the serum P1NP concentration (33.1% and 46.7%, respectively; *p* < 0.05 and *p* < 0.001, respectively) compared to that of the USN group. However, the serum CTX-1 in the USN group increased significantly four weeks after neurectomy compared to that of the Sham group (81.7%; *p* < 0.001), while treatment with 100 mg/kg and 300 mg/kg naringin resulted in a 35.6% and 47.8% decrease in the plasma CTX-1 level, respectively, compared to that of the USN group (*p* < 0.01 and *p* < 0.001, respectively; Fig. 3Ab). Both of these bone-turnover markers were at least restored to the levels of the sham group after treatment with higher doses of naringin, although there were no significant differences (both *p* > 0.05). The level of urinary Dpd increased significantly due to neurectomy in the USN group compared to that of the Sham group (*p* < 0.001), while the levels of urinary Dpd in the 100 mg/kg and 300 mg/kg naringin groups decreased by 32.9% and 32.8%, respectively, compared to those of the USN group (both *p* < 0.001; Fig. 3Ac). Based on the decreased bone formation marker plasma P1NP and increased bone resorption markers plasma CTX-1 and urinary Dpd, we demonstrate here a negative bone balance in the USN group due to elevated bone resorption and declining bone formation. In contrast, higher doses of naringin could enhance bone formation and decrease bone resorption, as indicated by the bone-turnover markers. Thus, naringin could enhance osteogenic activity and simultaneously inhibit osteoclast resorption activity, ultimately leading to increased bone mass.

Because BMD is also an important indicator of bone quality and strength, we assessed the BMD of distal femoral metaphysis among all of the groups. The BMD of the distal femur in the USN group decreased significantly compared to that of the sham-operated rats (15.7%; *p* < 0.01; Fig. 3Ad). However, after treatment with higher doses of naringin for eight weeks, the BMD of the distal femur increased by 15.6% and 17.5%, respectively, in the 100 mg/kg and 300 mg/kg naringin groups (both *p* < 0.05) compared to that of the USN group, returning to the levels of the Sham group (both *p* > 0.05).

### Mineral apposition rate (MAR)

To dynamically assess bone formation in the distal femoral metaphysis in each group, a dynamic histomorphometric analysis was performed by the intramuscular injection of tetracycline 10 and 3 days before sacrifice. Representative tetracycline fluorescence images of the distal femoral metaphysis in each group are shown in Fig. 3Ba, and the mineral apposition rate was analysed (Fig. 3Bb). MAR significantly decreased 4 weeks after neurectomy in the USN group, while the naringin-treated groups at higher doses had a higher MAR than that of the USN group. Thus naringin could prevent decreased bone mineralization caused by neurectomy.

### Expression of OCN and TRAP

**Figure 4:**
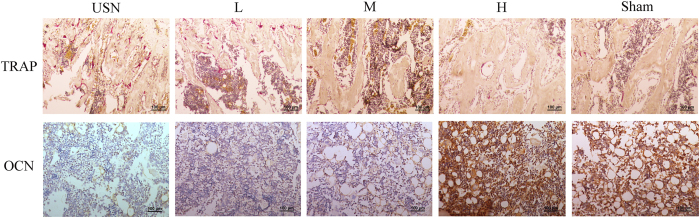
Effect of naringin on osteogenesis and osteoclastogenesis *in vivo* in each group. The osteoblastic activities in sections of the distal femoral metaphysis taken 4 weeks post-operation were indicated by the immunohistochemical staining of OCN (osteocalcin), and osteoclasts in the distal femoral metaphysis were indicated by TRAP staining. The immunohistochemical reactivity of OCN in the USN group was lower than that of the Sham group, while the OCN staining in the groups that were administered naringin was gradually enhanced. More TRAP-positive cells were observed in the USN group compared to the Sham group; this number significantly decreased in the naringin-treated groups at higher doses. Osteoclasts were indicated as TRAP-positive cells containing >2 nuclei and stained red on the surface of the trabecular bone. L represents the 30 mg/kg Naringin group, M the 100 mg/kg Naringin group, and H the 300 mg/kg Naringin group.

To simultaneously evaluate bone formation and osteoclast resorption within the bony tissue in each group, we measured the expression of OCN, a specific marker of osteogenic activity, and TRAP, an index for osteoclastogenesis, by the histological staining of the distal femurs of each group. As shown in Fig. 4, neurectomy dramatically decreased the immunohistochemical reactivity of OCN in bony tissue compared to that of the Sham group. With the increasing concentration of naringin, the immunohistochemical reactivity of OCN increased gradually; the 300 mg/kg naringin group displayed the greatest immunohistochemical reactivity. In contrast, the number of TRAP-positive osteoclasts on the surface of the trabecular bone in the USN group markedly increased compared to that of the Sham group, while naringin at a higher dose significantly decreased TRAP-positive osteoclasts compared to the USN group (Fig. 4). From the results above, it is hypothesized that naringin promotes bone formation and simultaneously inhibits bone resorption, thus increasing bone mass.

### TRAP staining and quantitatively measuring ALP activity *in vitro*

**Figure 5:**
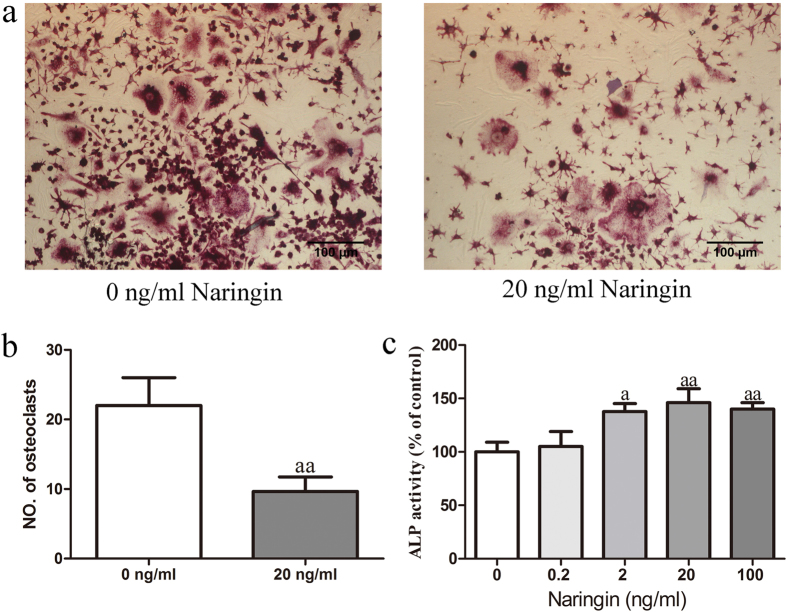
Effect of naringin on the differentiation of osteoclasts and osteoblasts *in vitro*. The differentiation of RAW 264.7 cells into osteoclasts was assessed by TRAP staining 7 days after naringin treatment. The differentiation of MC3T3-E1 cells into osteoblasts was evaluated using an ALP activity kit 7 days after naringin treatment. The dose of 20 ng/ml naringin dramatically decreased the number of TRAP-positive osteoclasts (**a**,**b**) and increased the ALP activities of osteoblasts in a dose-dependent manner; 20 ng/ml naringin showed the maximum effect (**c**). The experiments were performed in triplicate, n = 3 per experiment. ^a^*p* < 0.05, ^aa^*p* < 0.01, ^aaa^*p* < 0.001 vs. Sham group. ^b^*p* < 0.05, ^bb^*p* < 0.01, ^bbb^*p* < 0.001 vs. USN group. ^c^*p* < 0.05, ^cc^*p* < 0.01, ^ccc^*p* < 0.001 vs. 300 mg/kg Naringin group. Student’s t test for (**b**), One-way ANOVA for (**c**).

To further confirm the conclusion above that naringin has an anabolic effect on bone, we investigated the effect of naringin on osteoclast and osteoblast differentiation *in vitro* by TRAP staining and by evaluating ALP activity, respectively. The differentiation of RAW 264.7 cells into osteoclasts was induced with 100 ng/ml RANKL. Naringin at 20 ng/ml significantly decreased the number of mature osteoclasts compared to that of the group without naringin (Fig. 5a,b). Naringin significantly enhanced the activity of ALP, a marker of early-stage osteoblast differentiation, dose-dependently, with the maximal effect at 20 ng/ml (Fig. 5c). Therefore, we evidence both *in vivo* and *in vitro* that naringin promotes bone formation and simultaneously inhibits bone resorption.

### Expression of Sema3a, Nrp1 (neuropilin-1) and β-catenin mRNA

**Figure 6:**
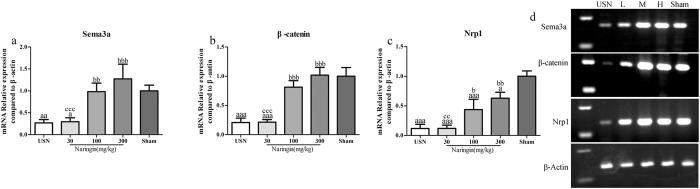
Effects of naringin on the mRNA expression of Sema3a, β-catenin and Nrp1. Four weeks following USN, the total RNA was collected from the tibias ipsilateral to USN in the different treatment groups and subjected to qRT-PCR. Sema3a (**a**), β-catenin (**b**) and Nrp1 (**c**) in the tibias ipsilateral to USN in each group were analysed by qRT-PCR corrected by β-Actin relative to the Sham group, and reverse transcribed (**d**). ap < 0.05, aap < 0.01, aaap < 0.001 vs. Sham group. bp < 0.05, bbp < 0.01, bbbp < 0.001 vs. USN group. cp < 0.05, ccp < 0.01, cccp < 0.001 vs. 300 mg/kg Naringin group. L represents the 30 mg/kg Naringin group, M the 100 mg/kg Naringin group, and H the 300 mg/kg Naringin group. One-way ANOVA. Full-length gels are presented in Supplementary Figure S1.

Sema3A is the first local determinant of bone mass in bony tissues by binding its receptor Nrp1 expressed on the membrane surface of both osteoblasts and osteoclasts. Both the increased expression of Sema3A and the overexpression of Nrp1 on the osteoclast surface led to the inhibition of RANKL-induced osteoclastic differentiation, and Sema3A binding with Nrp1 could stimulate the Wnt/β-catenin signalling pathway. β-Catenin is thus significantly translocates and accumulates in the cell nucleus, ultimately enhancing osteoblast differentiation^10^. In addition, the osteoprotective role of Sema3A is also mediated by its effect on maintaining normal sensory innervation into bone^11^. To investigate whether the Sema3A-stimulated Wnt/β-catenin signalling pathway is involved in the osteoprotective role of naringin, we first analysed by qRT-PCR the mRNA levels of Sema3a, together with its receptor Nrp1, and β-catenin in the tibias of all of the groups. As shown in Fig. 6a,d (also Supplementary Figure S1), four weeks after USN, the expression of Sema3a in ipsilateral tibias to USN decreased 3.69-fold compared to that of the sham-operated group (*p* < 0.01). In the naringin-treated groups, the mRNA of Sema3a increased 3.62-fold at 100 mg/kg naringin and 4.69-fold at 300 mg/kg compared to that of the USN group, with statistically significant differences (*p* < 0.05 and *p* < 0.001, respectively). For β-catenin, after USN, the expression of β-catenin mRNA decreased 4.81-fold compared to that of the Sham group (*p* < 0.01). With naringin treatment at 100 mg/kg and 300 mg/kg, the β-catenin mRNA expression increased significantly, 3.91 and 4.90-fold higher than that of the USN group, respectively (Fig. 6b,d and Supplementary Figure S1), and the difference is statistically significant (both *p* < 0.001). Further analysis of the qRT-PCR data demonstrated that naringin at 300 mg/kg could restore the reduced Sema3a and β-catenin levels to at or above the levels of the Sham group (*p* > 0.05).

As illustrated in Fig. 6c,d (also Supplementary Figure S1), the Nrp1 mRNA decreased 8.55-fold after USN compared to the Sham group (*p* < 0.001). In contrast, after treatment with 100 mg/kg naringin, the Nrp1 mRNA level increased 3.74-fold compared to that of the USN group (*p* < 0.05). In addition, 300 mg/kg naringin could even more strongly increase Nrp1 expression, which increased 5.38-fold compared to that of the USN group, with statistically significant differences (*p* < 0.01). However, the mRNA level of Nrp1 as increased by naringin was still lower than that of the Sham group (*p* < 0.05). Thus, we demonstrated at gene transcription levels that naringin could revere the immobilization-induced downregulation of Sema3A and the inactivation of the Wnt/β-catenin signalling pathway.

### Expression of Sema3A protein

**Figure 7:**
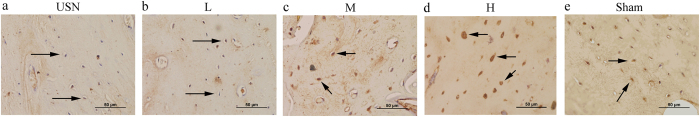
Sema3A expression in response to USN with or without naringin administration. (**a**) Immuno-histochemical staining of Sema3A in the femoral midshaft sections of rats from different treatments 4 weeks after USN showed decreased Sema3A-positive intensity in the osteocytes that were distributed in the femoral diaphysis of the USN rats but significant up-regulation in the naringin-treated rats, especially at higher doses of naringin. L represents the 30 mg/kg Naringin group, M the 100 mg/kg Naringin group, and H the 300 mg/kg Naringin group. Magnification ×400. Full-length blots are presented in Supplementary Figure S2.

**Figure 8:**
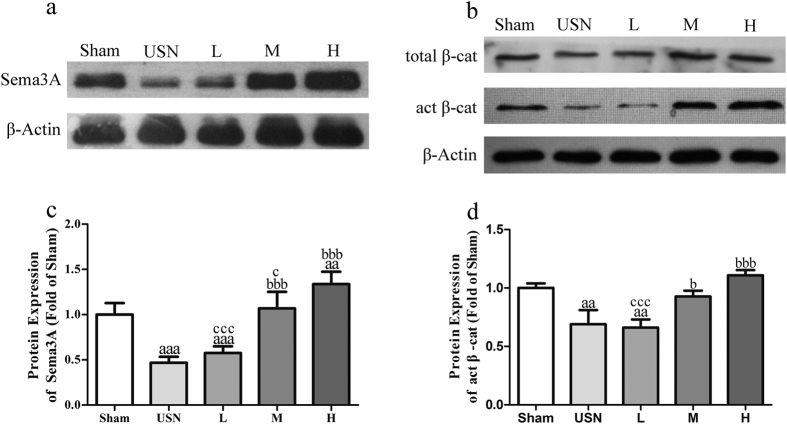
Effects of naringin on the expression of Sema3A and active and total β-catenin protein in each group. The total proteins that were extracted from the tibias ipsilateral to USN at the indicated times were analysed by western blotting for Sema3A (**a**) and active (act) and total β-catenin (**b**), with actin serving as the loading control. The band intensities as analysed by densitometry showed that the relative quantification of the expression of protein Sema3A (**c**) and active (act) (**d**) in the USN rats decreased dramatically compared to that of the Sham group and significantly increased at higher doses of naringin. ^a^*p* < 0.05, ^aa^*p* < 0.01, ^aaa^*p* < 0.001 vs. Sham group. ^b^*p* < 0.05, ^bb^*p* < 0.01, ^bbb^*p* < 0.001 vs. USN group. ^c^*p* < 0.05, ^cc^*p* < 0.01, ^ccc^*p* < 0.001 vs. 300 mg/kg Naringin group. L represents the 30 mg/kg Naringin group, M the 100 mg/kg Naringin group, and H the 300 mg/kg Naringin group. One-way ANOVA. Full-length blots are presented in Supplementary Figure S3.

We next examined the expression level of Sema3A at the translational level by immunohistochemistry and western blotting. Because Sema3a mRNA is predominantly expressed by osteoblasts and osteocytes, whereas Sema3a mRNA in osteoclasts is undetectable^10^, the Sema3A protein expression was first detected in osteocytes by the immunohistochemical staining of the diaphysis of femurs. As Fig. 7 illustrates, in the Sham group, Sema3A protein was expressed to a certain degree in the osteocytes of femurs. However, after USN, the immunostaining intensity of Sema3A in osteocytes decreased dramatically compared to that of the Sham group. However, after treatment with naringin, the staining intensity of Sema3A in osteocytes gradually increased, with the maximal effect observed in the 300 mg/kg naringin-treated group (Fig. 7). To confirm the immunohistochemical results, the expression of Sema3A protein in ipsilateral tibias was also assayed by western blot analysis. As shown in Fig. 8a,c (also Supplementary Figure S2), the concentration of Sema3A in the USN group was much lower than that in the Sham group. In addition, after the oral delivery of naringin to sciatic neurectomized rats, the expression of Sema3A in the tibias increased gradually with the increasing dose of naringin, with the maximum effect at 300 mg/kg naringin; the sema3A concentration was much higher than that of the USN group. Thus, we further confirmed at the protein level that naringin could promote the expression of Sema3A protein, which was significantly decreased by USN.

### Activation of the Wnt/β-catenin signalling pathway

Because osteocytes communicate with each other, as well as osteoblasts or osteoclasts, through canaliculi and dendritic connections, Sema3A secreted by osteocytes could be delivered to osteoblasts and osteoclasts to affect their biological activities. Previous studies revealed that Sema3A promotes osteoblast differentiation by activating the canonical Wnt/β-catenin signalling pathway^10^; thus, we then investigated the change in the active β-catenin protein and total β-catenin levels in the tibias of each group by western blotting. The Western blotting results showed that with the reduction of Sema3A protein expression in ipsilateral femurs and tibias after USN, the active β-catenin protein level in tibias ipsilateral to USN decreased significantly compared to that of the Sham group (Fig. 8b,d, also Supplementary Figure S3). In contrast, after the USN rats received naringin treatment, the concentration of active β-catenin increased gradually, with the highest level at 300 mg/kg naringin. However, the total β-catenin protein levels in the different groups were not obviously different. Thus, naringin could activate the Wnt/β-catenin signalling pathway in bony tissues.

## Discussion

Naringin has been extensively studied for its protective role in the maintenance of bone mass in various models of osteoporosis; however, no report has investigated the effect of naringin on disuse osteoporosis or its corresponding mechanism. Disuse osteoporosis is characterized by BMD decrease, bone microstructure disruption and mechanical strength deterioration due to musculoskeletal system unloading^30^,^31^,^32^. Because the deterioration of trabecular microarchitecture is difficult to fully restore, it is more important to prevent rather than treat osteoporosis^33^. Thereby, in our study, we emphasize the preventive role of naringin in the occurrence and progress of disuse osteoporosis. For the first time, we provide evidence that naringin plays a beneficial role in the prevention of disuse osteoporosis due to hind limb immobilization induced by USN. Kingery *et al.* demonstrated that following USN, the BMD in the ipsilateral cancellous bone of the femur and tibia decreased dramatically with significant difference, and the disuse of the hind limb ipsilateral to neurectomy was obvious, as the weight bearing ability decreased markedly^15^. Because the progress of disuse osteopenia mainly occurred during the first four weeks after USN^15^, we observed an effect of naringin on osteoporosis for four weeks following denervation.

Our BMD measurements showed that the distal femoral BMD in the USN group was much lower than that of the Sham group four weeks after neurectomy. However, the prophylactic application of naringin exhibited a pronounced beneficial role in bone mass maintenance dose dependently. The BMD of the distal femur in the naringin-treated groups at 100 mg/kg and 300 mg/kg was much higher than that of the USN group and was even restored to the level of the sham group. We further evaluated the biomechanical strength in each group, the results of which revealed that the maximal load, stiffness and energy absorption of the ipsilateral femoral neck to USN significantly decreased in the USN group compared to those of the sham group, while naringin treatment at a higher dose (100 mg/kg and 300 mg/kg) displayed a remarkably positive effect on the mechanical properties of the ipsilateral femoral neck. Because the maintenance of trabecular microstructure takes more responsibility for mechanical strength^34^, we then measured distal femoral trabecular microarchitecture in the different treatment groups. We found that the trabecular microarchitecture in the USN group was much more deteriorated, as indicated by the following trabecular parameters: the BV/TV, Tb.N, and Tb.Th in USN group significantly decreased, while the BS/BV and Tb.Sp dramatically increased compared to those of the sham group. In addition, all five of the above trabecular parameters significantly improved after oral administration with naringin at 100 mg/kg and 300 mg/kg compared to those of the USN group, even reaching the levels of the sham group.

We then measured the bone-turnover markers in each group and demonstrated that the biochemical marker of osteogenic activity P1NP in the serum significantly decreased four weeks after USN, while the markers of bone resorption plasmic CTX-1 and urinary Dpd increased markedly compared to those of the sham group, indicating that osteogenic activity decreases and bone resorption increases in disuse osteoporosis, in accordance with previous studies^14^,^35^. In contrast, the administration of naringin at higher doses (100 mg/kg and 300 mg/kg) increased serum P1NP but decreased serum CTX-1 and urinary Dpd, suggesting that naringin could promote bone formation while inhibiting bone resorption. This function of naringin in bone remodelling was also verified in bony tissues, as the immunohistochemistry of the osteoblast differentiation marker osteocalcin and the osteoclastic differentiation marker TRAP in the distal femoral metaphysis showed that the osteoblastic activity decreased, the osteoclastic activities increased in bony tissue under unloading in the USN group, and naringin at higher doses promoted osteoblast differentiation and weakened osteoclast differentiation *in vivo*. The direct roles of naringin in enhancing osteoblast differentiation and inhibiting osteoclastogenesis were further identified and confirmed *in vitro* by TRAP staining and by measuring ALP activity, respectively. In addition, previous studies also showed that naringin could promote osteoblast differentiation and up-regulate plasmic osteocalcin^22^, while inhibiting osteoclastogenesis and down-regulating plasmic CTX-1 in ovariectomized rodents^21^. Thus, this dual regulative role of naringin in bone remodelling demonstrates that naringin could be a potent anabolic agent in bone metabolism. Traditional drugs for osteoporosis target only bone formation or bone resorption^36^. However, bone remodelling couples bone formation and resorption; moreover, osteoblasts and osteoclasts, which are, respectively, responsible for the two processes, communicate and interact with each other^37^. RANKL secreted by osteoblasts promotes osteoclast differentiation^38^, and failure in osteoclastogenesis ameliorates bone formation^39^, meaning that the disruption of the activities of one of the two cell lineages always leads to the dysfunction of the other cell lineage. For example, the antiresorptive drug bisphosphonate also plays an inhibitory role in osteogenesis^40^, and anabolic agent PTH simultaneously promotes bone resorption^8^, both of which potentially risk aggravating osteoporosis, even leading to bone fracture; as a solution, combinative treatment is more effective in the enhancement of bone quality^41^. Therefore, the simultaneous antiresorptive and anabolic role of naringin shows great value in clinical disused osteoporosis treatment.

Because naringin plays a positive role in increasing bone formation and synchronously inhibits bone resorbing, we analysed the dynamic microstructural parameter mineral apposition rate (MAR), a dynamic parameter that is commonly used to evaluate the mean rate of new bone formation, by the tetracycline fluorescence double-labelling of undecalcified histologic sections of the distal femur of each group. Under a fluorescence microscope, we observed two fluorescent lines on the surface of the trabecular, one almost buried in the bone matrix and the other closer to the bone surface, which were marked by an intramuscular injection of tetracycline in rats at different times before the samples were harvested. By calculating the mean vertical distance between the two fluorescence lines, which was then divided by the marking time interval, we could obtain MAR (μm/d). An analysis of the results indicated that the ability of bone mineralization and bone formation in the USN group significantly decreased compared to that of the Sham group. However, naringin at higher doses could markedly promote the rate of bone formation.

Recent studies have reported that naringin could be a ligand-activating oestrogen receptor^27^. Oestrogen could be an antiresorptive agent^42^, but its role in activities of osteoblasts is still not clear^43^; thus dual role of naringin in bone remodelling, especially during disuse osteoporosis, cannot be attributed only to oestrogen-like action, the mechanism of which is far from clear. Sema3A is the first identified local factor in the bony microenvironment that exerts both a simulative role in osteoblastic bone forming and an inhibitive role in osteoclastic bone resorption^36^, independent of the classical OPG-RANKL-RANK signal axis^10^. Because naringin and Sema3A have similar dual roles in bone remodelling, we further investigated whether Sema3A signalling was implicated in the osteoprotective effect of naringin. With consistent results from qRT-PCR and Western blot analyses, the expression of Sema3A in the tibias ipsilateral to USN significantly decreased compared with to that of the sham group, and naringin at higher doses showed an ability to increase Sema3A expression in USN rats. The immunohistochemical staining of Sema3A confirmed that Sema3A-positive osteocytes showed a much higher immunoreaction intensity at higher doses of naringin than did both the USN group and the sham group. Because osteoblasts and osteocytes are the primary bone cell lineages secreting Sema3A^10^, we propose that naringin may promote osteocytes secreting Sema3A, which is then delivered to osteoblasts, osteoclasts and other osteocytes through canaliculi or dendritic connections, thus promoting osteoblast activities, inhibiting osteoclast activities and, in general, preventing bone loss induced by unloading. In addition, decreased Sema3A is correlated with the development of disuse osteoporosis. Because neuropilin-1 (Nrp1) exists in both osteoblasts and osteoclasts and mediates the effect of Sema3A^10^, we also measured the mRNA expression level of Nrp1 in the tibia, which significantly decreased due to denervation but significantly increased with naringin administration. We hypothesize that this expression pattern occurs in response to the increased expression of Sema3A. However, whether the increased expression of Nrp1 is in osteoblasts, osteoclasts, or both remains to be further investigated.

The activation of the Wnt/β-catenin signalling pathway protects β-catenin from being phosphorylated; thus, the unphosphorylated and activated β-catenin translocates and accumulates in the nucleus from the cytoplasm, which then binds with T cell factor/lymphoid enhancer factor (TCF/LEF)^44^, forming a transcription factor complex to enhance the expression of osteoblastic-specific genes. Thereby, Wnt/β-catenin signalling plays an essential role in bone formation^45^. Sema3A is an activator of the Wnt/β-catenin signalling pathway by binding to Nrp1 to induce osteoblast differentiation^10^. Naringin promotes β-catenin accumulation in the nucleus in osteoblast-like cells^46^. Because β-catenin is a vital molecule in Wnt/β-catenin signalling cascades, we next explored the levels of activated and total β-catenin in tibias ipsilateral to USN. qRT-PCR analysis demonstrated that β-catenin mRNA expression decreased after denervation but increased after naringin treatment at higher doses. In addition, the western blot results further revealed that the activated β-catenin protein level in the USN group was much less than that in the sham group. Oral administration with a higher dose of naringin significantly increased the activated β-catenin protein within bone tissues under an environment of immobilization compared to the USN group. These results indicate that the attenuation of Wnt/β-catenin signalling pathway activation is involved in the development of disuse osteoporosis. In addition, treatment with naringin could up-regulate activated β-catenin expression in the skeleton, which may at least partly be mediated by the up-regulation of Sema3A and may ultimately protect bone mass against lost induced by unloading.

Another recent study of the role of Sema3A in bone mass indicated that level of Sema3A in the bony microenvironment was co-determined by bone cells and sensory nerve system innervating bone, as both mice in which Sema3A was specifically knocked down in osteoblasts and mice with a specific deficiency of Sema3A in neurons exhibited reduced sensory innervation into bone and had less Sema3A expression in bony skeleton than that of wild-type mice^11^. Because the sciatic nerve is a source of peripheral nerve fibres innervating femoral and tibial cancellous bone^15^, the sciatic neurectomy in our model very likely leads to a reduction of ipsilateral femoral and tibial innervation, which may subsequently contribute to the decreasing Sema3A level in bony microenvironment in addition to bone cells. In addition, mice with a specific deficiency of Sema3A in osteoblasts had a normal bone mass *in vivo*; in contrast, mice with Sema3A knock-out specifically in neurons had an obvious reduction of bone mass^11^. These results suggest that the effect of Sema3A on bone homeostasis *in vivo* occurs mainly by controlling sensory innervation into bone indirectly and not by its direct role in bone cell activities. Therefore, in light of the essential role of the sensory nerve system in bone homeostasis, there is a possibility that naringin may exert its role by increasing sensory innervation into bone during the prevention of bone loss induced by immobilization. In addition, increasing the level of Sema3A in bone may be a sign of sensory reinnervation into bone or may mediate the effect of naringin in promoting sensory innervation. This hypothesis should be further investigated by the immunostaining of sensory nerve fibres in bones before and after immobilization, with or without naringin treatment. However, Mikihito *et al.* reported that the systemic administration of naringin *in vivo* could increase the bone mass of normal adult mice, prevent bone loss in ovariectomized mice and even accelerate the regeneration of bone defects by the local delivery of naringin. Thus, we believe that systemic or local increases of Sema3A can increase bone mass by synchronously promoting osteoblastic bone formation and by hampering osteoclastic bone resorption, although it is not ruled out that the bone anabolic effect of Sema3A is mediated by its role in regulating sensory innervation in bone. In addition, naringin promotes the expression of Sema3A in bone. In future studies, it is necessary to adopt another disuse osteoporosis model by hind limb suspension to test the conclusions above to reduce the influence of denervation or to administer naringin to normal animals to observe the change in sensory innervation in bone.

In conclusion, the osteoprotective role against disuse osteoporosis of naringin may be mediated as follows: 1) Naringin promotes the secretion of Sema3A by osteoblasts and osteocytes. Increasing Sema3A locally enhances osteoblastic bone formation via the activation of the Wnt/β-catenin signalling pathway and simultaneously inhibits osteoclastogenesis. 2) Naringin plays a direct role in activating the Wnt/β-catenin signalling pathway, thus inducing osteoblast differentiation. 3) Naringin facilitates sensory innervation in bone, which may be mediated by Sema3A. Our study demonstrates for the first time that the Sema3A-NRP1-β-catenin signalling axis plays a regulatory role in the development of disuse osteoporosis, through which naringin could be a promising therapeutic agent in the clinical prevention of disuse osteoporosis.

## Additional Information

**How to cite this article**: Ma, X. *et al.* Naringin ameliorates bone loss induced by sciatic neurectomy and increases Semaphorin 3A expression in denervated bone. *Sci. Rep.* **6**, 24562; doi: 10.1038/srep24562 (2016).

## Supplementary Material

Supplementary Information
