# Supplementary material for: Naringin ameliorates bone loss induced by sciatic neurectomy and increases Semaphorin 3A expression in denervated bone
Source: Sci Rep. 2016 Apr 25;6:24562. doi: 10.1038/srep24562 (PMC4842995; doi:10.1038/srep24562)
Supplement: Supplementary Information [file srep24562-s1.pdf]

Naringin ameliorates bone loss induced by sciatic neurectomy and increases Semaphorin 3A expression in denervated bone

Xinlong Ma[1,2,3,#,*], Jianwei Lv[1,2,3,#], Xiaolei Sun[1,2], Jianxiong Ma[1,2], Guosheng Xing[1], Ying Wang[1], Lei Sun[1], Jianbao Wang[1], Fengbo Li[1], Yanjun Li[1], Zhihu Zhao[1,2]

[1]Institute of Orthopaedics, Tianjin Hospital, No. 122, Munan Road, Heping District, Tianjin TJ 300050, China

[2]Tianjin Institute of Orthopaedics in Traditional Chinese and Western Medicine, No. 122, Munan Road, Tianjin TJ 300050, China

[3]Graduate School of Tianjin Medical University, No. 22, Qixiangtai Street, Heping District, Tianjin TJ 300070, China

*Address correspondence to:

Xinlong Ma, Institute of Orthopedics, Tianjin Hospital,

No. 122, Munan Road, Heping District, Tianjin TJ 300050, China.

E-mail：maxinlong8686@sina.com.

SUPPLEMENTARY INFORMATION includes:

Supplementary Figures S1-S3

a

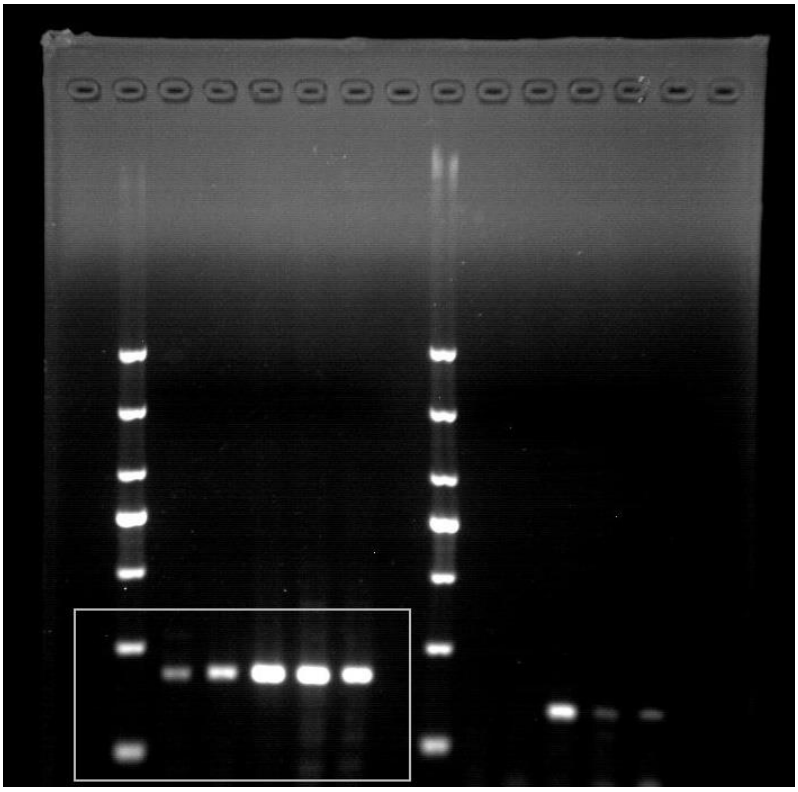

Sema3a

USN L M H Sham

b

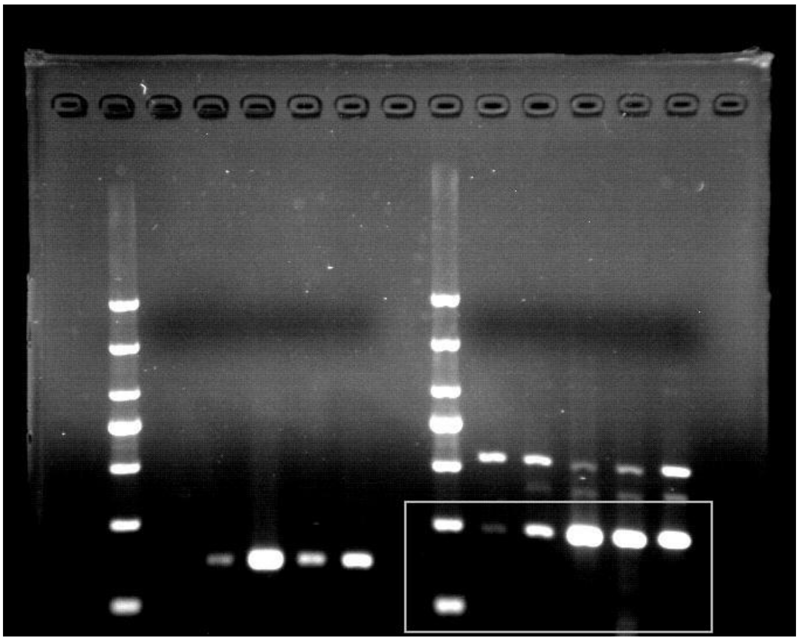

β-catenin

USN L M H Sham

c

Nrp1

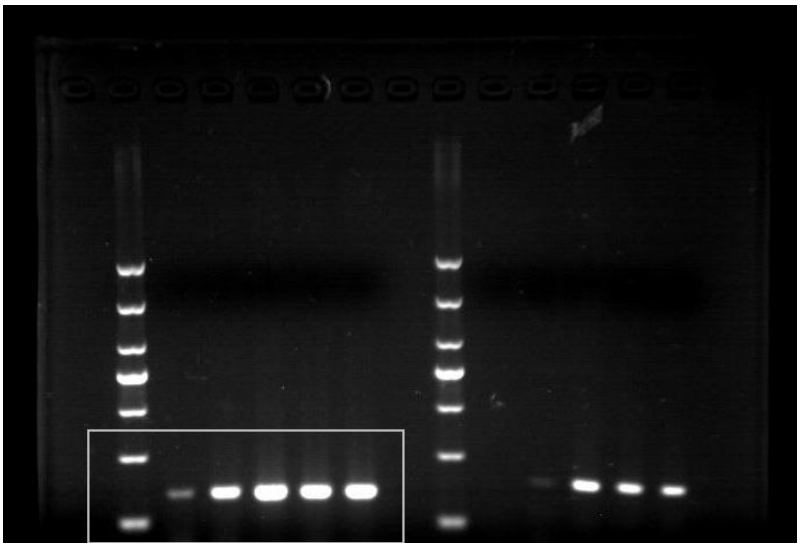

USN L M H Sham

d

β-Actin

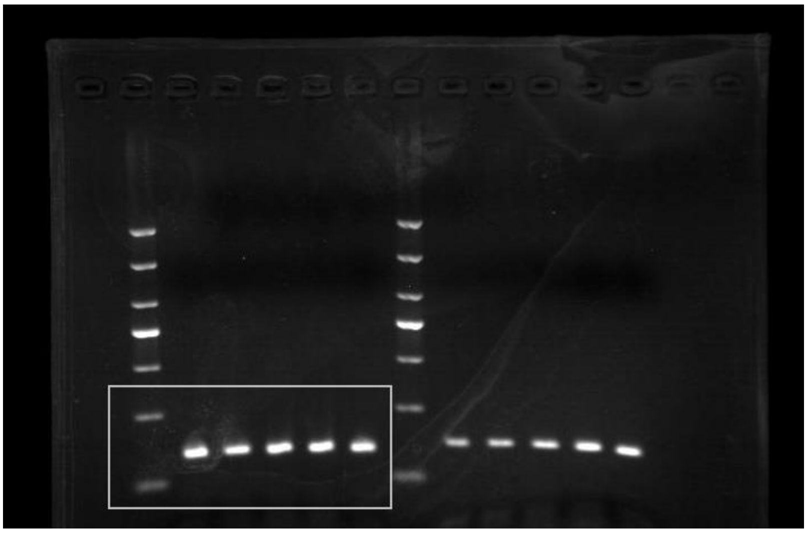

USN L M H Sham

Supplementary Figure S1: RT-PCR showing the different Sema3a (a), β-catenin (b) and Nrp1 (c) levels in each group. Full-length gels of Figure 6(d). USN represents unilateral sciatic neurectomy, L represents the 30 mg/kg Naringin group. M the 100 mg/kg Naringin group, and H the 300 mg/kg Naringin group. Sema3a is the abbreviation of Semaphorin3a, and Nrp1 is the abbreviation of neuropilin-1.

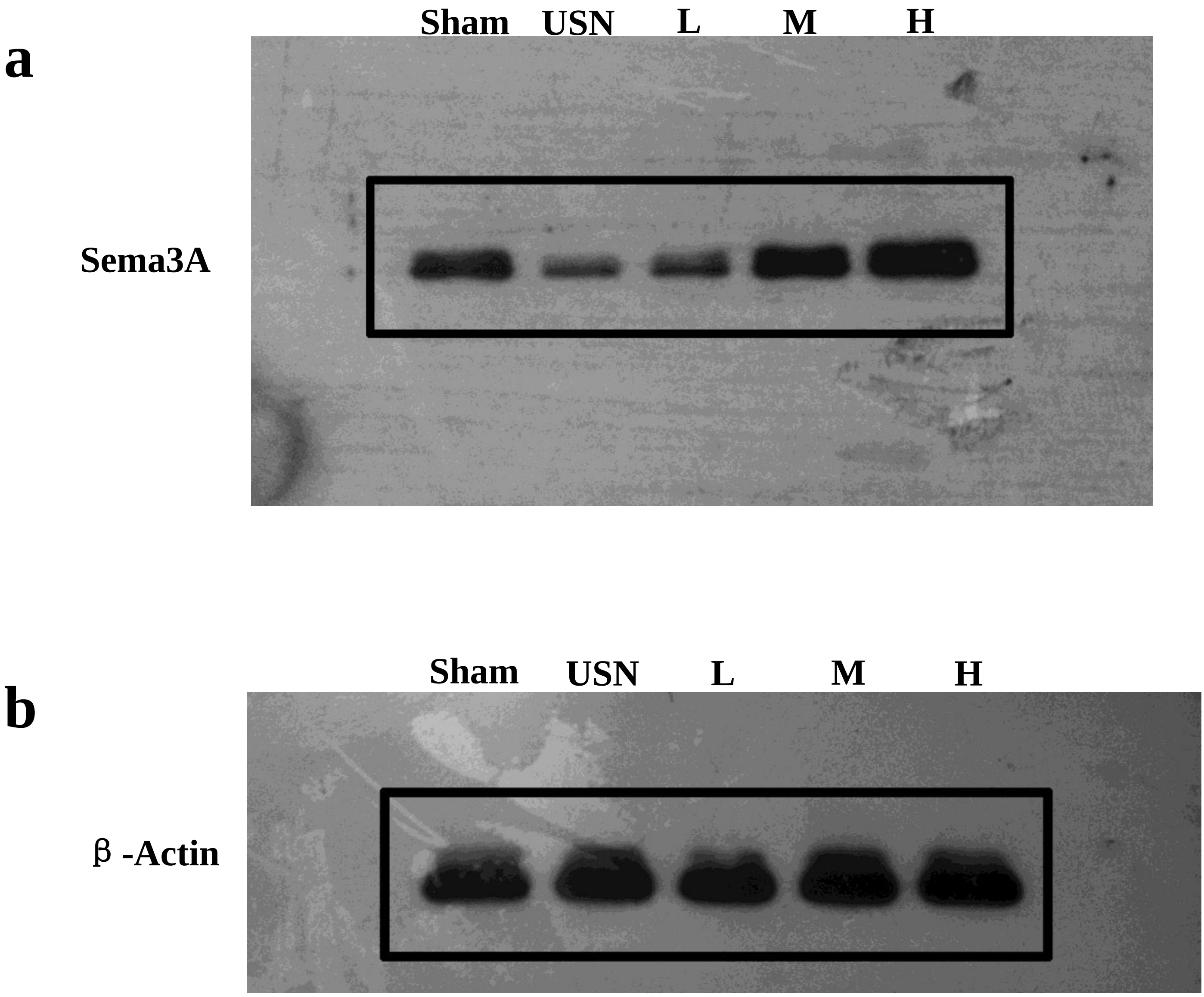

Supplementary Figure S2: Western blots showing the different Sema3A levels in each group. Full-length blots of Figure 8(a). USN represents unilateral sciatic neurectomy, L represents the 30 mg/kg Naringin group. M the 100 mg/kg Naringin group, and H the 300 mg/kg Naringin group. Sema3A is the abbreviation of Semaphorin3A.

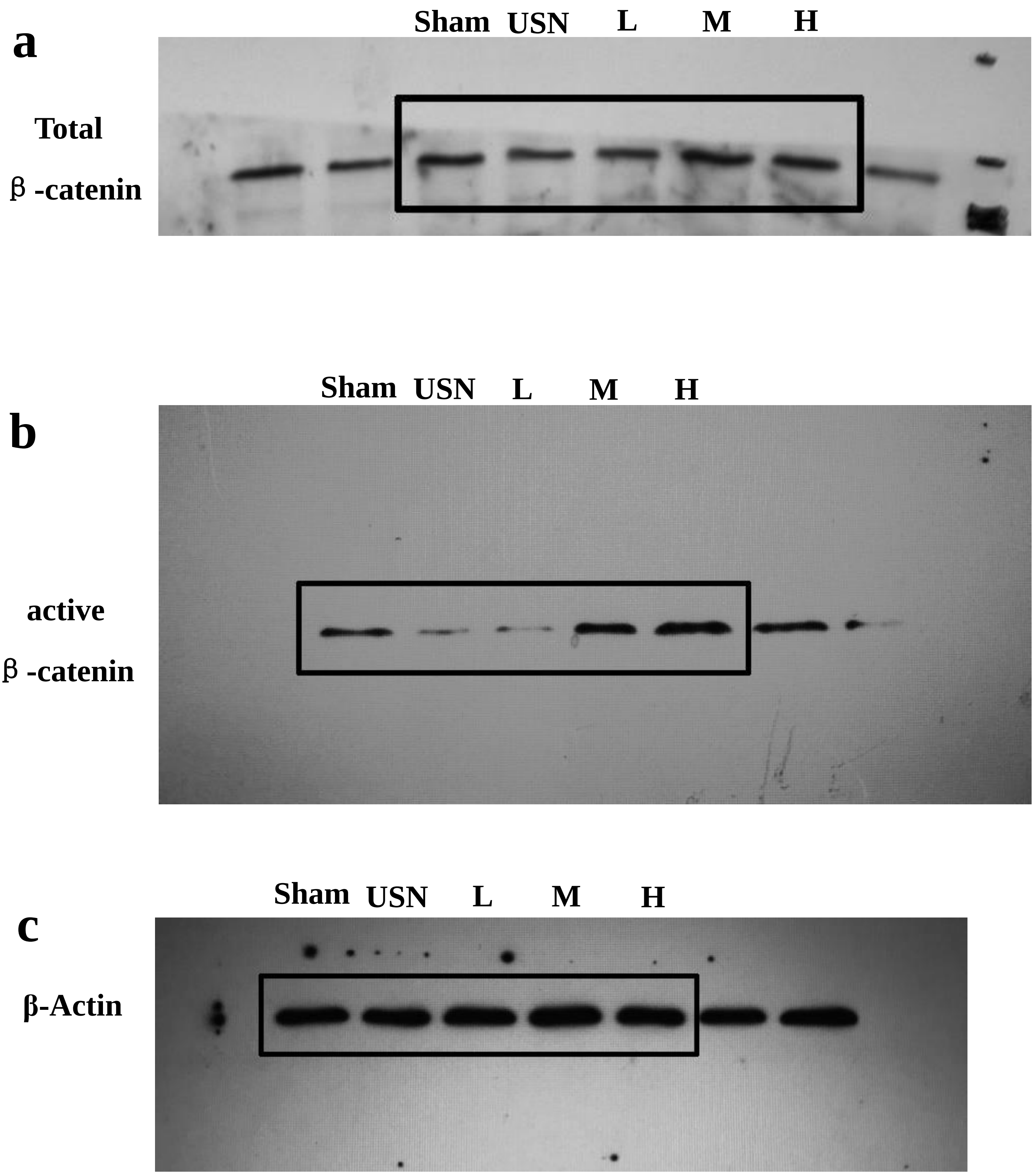

Supplementary Figure S3: Western blots showing the different total and active β-catenin levels in each group. Full-length blots of Figure 8(b). USN represents unilateral sciatic neurectomy, L represents the 30 mg/kg Naringin group. M the 100 mg/kg Naringin group, and H the 300 mg/kg Naringin group.
